# Topoisomerase II Is Required for the Proper Separation of Heterochromatic Regions during *Drosophila melanogaster* Female Meiosis

**DOI:** 10.1371/journal.pgen.1004650

**Published:** 2014-10-23

**Authors:** Stacie E. Hughes, R. Scott Hawley

**Affiliations:** 1 Stowers Institute for Medical Research, Kansas City, Missouri, United States of America; 2 Department of Molecular and Integrative Physiology, University of Kansas Medical Center, Kansas City, Kansas, United States of America; The University of North Carolina at Chapel Hill, United States of America

## Abstract

Heterochromatic homology ensures the segregation of achiasmate chromosomes during meiosis I in *Drosophila melanogaster* females, perhaps as a consequence of the heterochromatic threads that connect achiasmate homologs during prometaphase I. Here, we ask how these threads, and other possible heterochromatic entanglements, are resolved prior to anaphase I. We show that the knockdown of *Topoisomerase II* (*Top2*) by RNAi in the later stages of meiosis results in a specific defect in the separation of heterochromatic regions after spindle assembly. In *Top2* RNAi-expressing oocytes, heterochromatic regions of both achiasmate and chiasmate chromosomes often failed to separate during prometaphase I and metaphase I. Heterochromatic regions were stretched into long, abnormal projections with centromeres localizing near the tips of the projections in some oocytes. Despite these anomalies, we observed bipolar spindles in most *Top2* RNAi-expressing oocytes, although the obligately achiasmate *4^th^* chromosomes exhibited a near complete failure to move toward the spindle poles during prometaphase I. Both achiasmate and chiasmate chromosomes displayed defects in biorientation. Given that euchromatic regions separate much earlier in prophase, no defects were expected or observed in the ability of euchromatic regions to separate during late prophase upon knockdown of *Top2* at mid-prophase. Finally, embryos from *Top2* RNAi-expressing females frequently failed to initiate mitotic divisions. These data suggest both that Topoisomerase II is involved in the resolution of heterochromatic DNA entanglements during meiosis I and that these entanglements must be resolved in order to complete meiosis.

## Author Summary

Proper chromosome segregation during egg and sperm development is crucial to prevent birth defects and miscarriage. During chromosome replication, DNA entanglements are created that must be resolved before chromosomes can fully separate. In the oocytes of the fruit fly *Drosophila melanogaster*, DNA entanglements persist between heterochromatic regions of the chromosomes until after spindle assembly and may facilitate the proper segregation of chromosomes during meiosis. Topoisomerase II enzymes can resolve DNA entanglements by cutting and untwisting tangled DNA. Decreasing Topoisomerase II (Top2) levels in the ovaries of fruit flies led to sterility. RNAi knockdown of the *Top2* gene in oocytes resulted in chromosomes that failed to fully separate their heterochromatic regions during meiosis I and caused oocytes to arrest in meiosis I. These studies demonstrate that the Top2 enzyme is required for releasing DNA entanglements between homologous chromosomes before the onset of chromosome segregation during *Drosophila* female meiosis.

## Introduction

In most organisms, crossing over between homologs during meiosis ensures their faithful segregation at the first meiotic division. However, in *Drosophila melanogaster* females, the *4^th^* chromosomes are always achiasmate, and the *X* chromosomes normally fail to crossover in 6–10% of oocytes [1]. Nonetheless, *Drosophila* females can segregate these achiasmate chromosomes with high efficiency, demonstrating the existence of a system (termed the distributive system) to segregate homologous chromosomes that fail to recombine [2].

Heterochromatic regions on the achiasmate chromosomes are both necessary and sufficient for the proper segregation of achiasmate homologs, and homologous heterochromatic regions remain tightly paired throughout prophase of *Drosophila* female meiosis [3]–[5]. However, during prometaphase I, achiasmate chromosomes move dynamically on the spindle before properly biorienting and then congress into a mass with the chiasmate chromosomes at metaphase I [6], [7]. During these movements, achiasmate *4^th^* and *X* chromosomes are connected by heterochromatic threads, which may play a role in the mechanism by which heterochromatin mediates chromosome segregation [6], [8]. How these threads are formed is unknown, but they could potentially arise from stalled replication intermediates [9].

Evidence for such connections between segregating meiotic chromosomes was first observed in crane fly spermatocytes [10]. Cutting the centromere from the arm of a segregating anaphase I chromosome led to re-association of the severed arm with its homolog on the opposite half-spindle, supporting the idea that chromosomes are able to maintain physical connections during meiosis I and that these connections can generate the force necessary to bring chromosomal regions together [10]. Additionally, chromosomal associations were observed during anaphase I in *D. melanogaster* sperm mutant for components of the condensin complex [11]. These studies indicate that the thread-like structures connecting chromosomes may be a conserved mechanism for segregating meiotic chromosomes.

To prevent loss of genetic material, such connections between homologs need to be resolved before anaphase I. Topoisomerase II enzymes are capable of creating double-strand breaks in DNA to resolve DNA entanglements during replication and transcription [12]. This function makes topoisomerase II or topoisomerase II-like enzymes possible candidates for resolving heterochromatic DNA threads between homologs during meiosis. Unfortunately, the study of topoisomerase II enzymes has been limited in meiosis due to the requirement of these enzymes to resolve DNA concatenations caused by replication in mitosis, as well as other potential roles in recombination, transcription and chromosome condensation [12]. Thus, most strong loss-of-function mutations of topoisomerase II enzymes are lethal. Examining the function of topoisomerase II during meiosis is further complicated by the presence of two topoisomerase II enzymes in many organisms.

Various studies have tried to address these issues either by chemically inhibiting topoisomerase II enzymes, such as in mice [13]–[16], or by induction of conditional mutations of *topoisomerase 2 (top2)*, as in yeast [17]–[19]. Shifting a temperature-sensitive *top2* mutant of *Saccharomyces cerevisiae* to the restrictive temperature during meiosis led to arrest just prior to spindle assembly [19]. Shifting a temperature-sensitive *top2* mutant of *Schizosaccharomyces pombe* to the restrictive temperature during meiosis led to arrest during the first meiotic division [17]. In both yeasts, earlier stages of meiosis appeared normal at restrictive temperatures [17]–[19]. Additionally, blocking recombination in the *top2* mutants of both types of yeast resulted in yeast that could progress past their initial arrest but were still unable to successfully complete meiosis II [17], [18]. Based on these studies, it was concluded for both types of yeast that Top2 was required to resolve DNA entanglements that form between recombinant homologs at meiosis I [17], [18].

*D. melanogaster* contains only a single gene encoding a topoisomerase II enzyme, *Top2*, and null mutations in *Top2* in *Drosophila* are lethal [20]. Heterozygous combinations of weak loss-of-function mutations are viable in some cases, but ovarian development is either so severely disrupted to prevent analysis or the mutations only mildly decrease Top2 function and/or protein levels [21]. These factors have made it difficult to fully assess the role that Top2 plays in the resolution of heterochromatic DNA threads at later stages of *Drosophila* female meiosis [21].

A solution to these difficulties was created when the Transgenic RNAi Project (TRIP) at Harvard Medical School made available a *Top2* RNAi-expressing line that is inducible in the female germline using the maternal alpha-tubulin GAL (matαGAL) driver [22]. The matαGAL driver appears to start expressing robustly at approximately stage 3 of the *Drosophila* ovary [23]. By expressing *Top2* RNAi with the matαGAL driver, Top2 levels can be decreased after the completion of the ovarian mitotic divisions where Top2 is essential and after the initiation of recombination where Top2 could potentially play a role. We will show that *Top2* RNAi expression during prophase of *Drosophila* female meiosis leads to specific defects in the ability of heterochromatic regions to fully separate and for the achiasmate *4^th^* chromosomes to move precociously towards the spindle poles in prometaphase I, despite the completion of spindle assembly. More importantly, we will demonstrate that the defect in chromosome separation leads to the inability of oocytes to successfully complete meiosis I, illustrating that the separation of heterochromatic regions is essential for the proper completion of meiosis.

## Results

### *Top2* RNAi oocytes exhibit abnormal chromosomal projections during prometaphase I

Because strong loss-of-function alleles of *Top2* are lethal [20], an RNAi construct targeting the *Drosophila Top2* gene was expressed using the maternal α-tubulin GAL driver ({matalpha4-GAL-VP16}V37 or matαGAL for short) [22]. This allowed us to examine the effect of decreased Top2 levels starting at approximately stage 3 of the ovary during mid-prophase [23]. By Western blot, the level of Top2 protein present in *Top2* RNAi/*matαGAL* oocytes was reduced compared to control lines, including flies heterozygous for only the *matαGAL* driver or only the *Top2* RNAi construct (Figure S1). The knockdown was not complete, as a weak band of Top2 was typically present.

**Figure 1:**
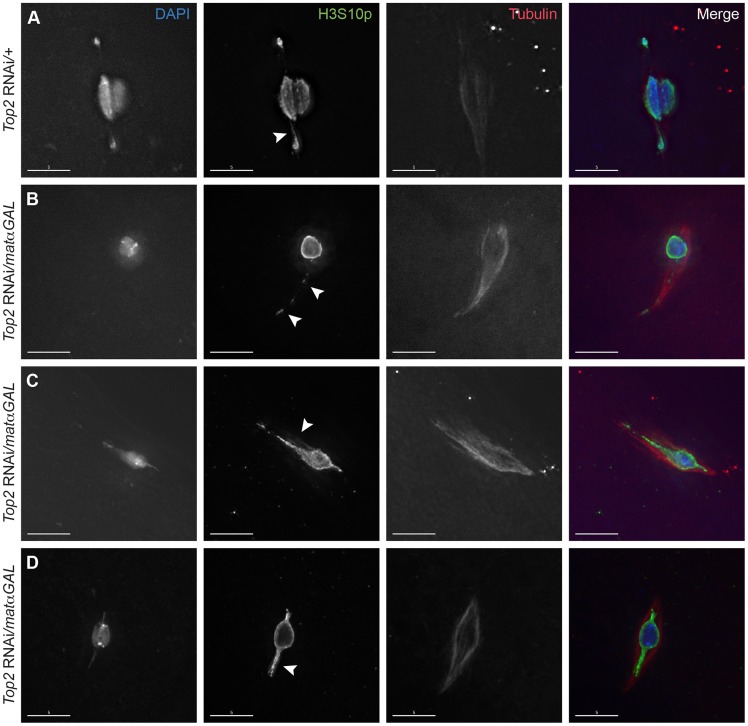
Expression of a *Top2* RNAi construct in the ovary leads to abnormal chromosomal projections during meiosis I. DNA is labeled with DAPI (blue), chromatin is labeled with an antibody recognizing H3S10p (green), and the spindle is labeled with an antibody recognizing α-tubulin (red). (A) *Top2* RNAi*/+* control oocyte with achiasmate *4^th^* chromosomes that have moved towards the spindle poles. Arrowhead points to the H3S10p-labeled heterochromatic DNA thread emanating from the achiasmate *4^th^* chromosome. (B–D) Shown are examples of *Top2* RNAi*/matαGAL* oocytes containing abnormal DNA projections that do not appear to connect chromosomes (arrowheads). Images are projections of partial Z-stacks. Scale bars are 5 microns.

We analyzed *Top2* RNAi-expressing oocytes using immunofluorescence with an antibody recognizing α-tubulin to mark the meiotic spindle and one recognizing histone 3 phosphorylated at serine 10 (H3S10p), which marks nuclei that have entered prometaphase I of meiosis and fortuitously highlights the DNA threads connecting achiasmate chromosomes during *D. melanogaster* female meiosis [8]. During prometaphase I in wild-type oocytes, achiasmate chromosomes biorient, and the obligately achiasmate *4^th^* chromosomes move toward opposite poles of the bipolar spindle. H3S10p-positive threads are frequently observed emanating from, and often connecting, these chromosomes (Figure 1A) [8]. The achiasmate chromosomes then congress back to the chiasmate chromosomes by metaphase I and the chromosome mass forms a lemon-shaped structure [7].

Preparations of oocytes from two-day-post-eclosion mated females are enriched for prometaphase I oocytes [7]. Achiasmate homologs (usually the *4^th^* chromosomes) were observed fully separated from the main chromosome mass in 40.0% (10/25) of such prometaphase I-enriched oocytes from mothers bearing only the *Top2* RNAi construct (*Top2* RNAi*/+*) and in 40.7% (11/27) of oocytes from mothers bearing only the driver (*matαGAL/+*). However, in similar preparations of *Top2* RNAi/*matαGAL* oocytes, achiasmate chromosomes were rarely observed completely separated from the chiasmate chromosomes (3.7% of oocytes, 1/27). In addition, in 44.4% (12/27) of *Top2* RNAi/*matαGAL* oocytes, one or more DNA projections were present that extended toward the spindle poles, with three of these oocytes containing two projections (Figure 1B–D). These projections stained positive for H3S10p and did not appear to connect chromosomes (Figure 1B–D). Similar projections were not observed in control oocytes heterozygous for only the *Top2* RNAi construct or the *matαGAL* driver (N = 25 and 27, respectively). Thus, in *Top2* RNAi/*matαGAL* oocytes, achiasmate homologs fail to separate from the main chromosome mass, as is observed in control oocytes. Rather, we often observe abnormal DNA projections emanating from that mass toward the pole. The nature of these projections is discussed below.

### Abnormal DNA projections in *Top2* RNAi*/matαGAL* oocytes contain centromeres

**Figure 2:**
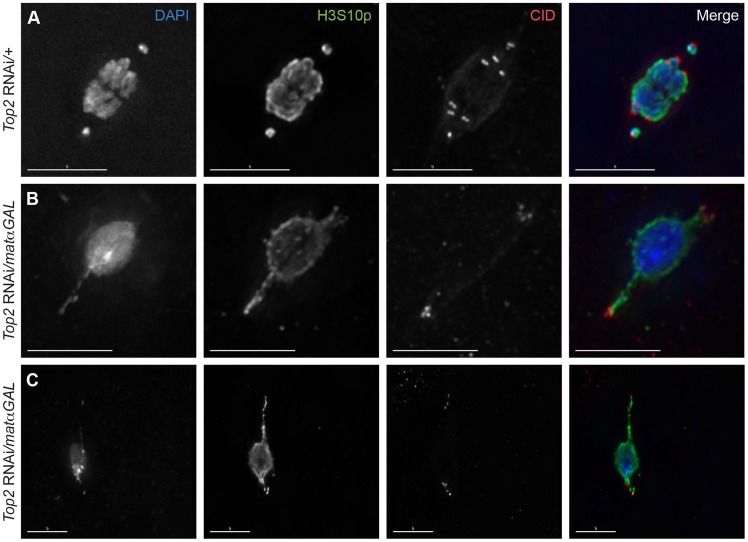
Centromeres are present within and near the tips of the DNA projections in *Top2* RNAi*/matαGAL* oocytes. DNA is labeled with DAPI (blue), chromatin is labeled with an antibody recognizing H3S10p (green), and centromeres are labeled with an antibody recognizing CID (red). (A) *Top2* RNAi/*+* control oocyte with achiasmate *4^th^* chromosomes that have moved towards the spindle poles with all eight centromeres properly bioriented. (B–C) Examples of *Top2* RNAi*/matαGAL* oocytes with abnormal DNA projections containing CID foci at or near the tips of the projections, indicating that the centromeres of multiple chromosomes have moved towards the spindle poles. Images are projections of partial Z-stacks. Scale bars are 5 microns.

The location of centromeres in relationship to the DNA projections was assessed using an antibody recognizing Centromere Identifier (CID), the *Drosophila* CENP-A homolog [24]. In wild-type prometaphase I oocytes, a CID focus is typically observed leading each achiasmate *4^th^* chromosome that has moved toward the spindle poles, while the six CID foci of the chiasmate chromosomes are located within the chromosomal mass such that homologous centromeres are oriented toward opposite spindle poles (Figure 2A). In *Top2* RNAi-expressing oocytes, CID foci could be observed at or near the tip of the DNA projections in 97.5% (39/40) of projections analyzed, indicating that centromere-led movements of the chromosomes towards the spindles poles are the cause of the DNA projections (Figure 2B, C).

More than one CID focus could be seen within some projections (Figure 2B, C), with five foci being the maximum number observed within a single projection within one oocyte. In all but four oocytes examined, the CID foci displayed some degree of clustering, making acquiring an accurate average number of CID foci within the projections impossible. However, Figure 2B shows an example where all the CID foci appear to be present within the two projections. This finding suggests that the projections are not simply the *4^th^* chromosomes becoming entangled with the autosomes and stretching as they attempt to move toward the poles, but that centromeres of chiasmate chromosomes are also moving toward the poles in some *Top2* RNAi*/matαGAL* oocytes. These results demonstrate that decreased *Top2* levels generally affect the movement and organization of centromeres within the chromosome mass. The data also suggest that the DNA protrusions are caused by the centromeres of both achiasmate and chiasmate chromosomes being pulled or pushed towards the spindle poles while other parts of the chromosomes are still anchored at the center of the spindle. Such movements would result in portions of the chromosomes being stretched out behind the centromeres.

### *Top2* RNAi/*matαGAL* oocytes form bipolar spindles

To determine whether defects in spindle assembly were the cause of the abnormal centromere–led DNA projections, we examined spindle morphology in *Top2* RNAi/*matαGAL* and control oocytes. In *Top2* RNAi*/+* oocytes, 87.5% (21/24) of spindles were bipolar and 75.0% (18/24) were tapered at both ends of the spindle. In *matαGAL/+* oocytes, 100% (27/27) of spindles were bipolar and 74.0% (20/27) were fully tapered at both ends. Despite the abnormal DNA projections described above, chromosomes in *Top2* RNAi*/matαGAL* oocytes were also able to organize a bipolar spindle in 88.5% (23/26) of oocytes, though in some cases one or both sides of the spindle were elongated to accommodate the chromosomal projections (Figure 1B–D). However, tapering of both ends of the spindle was only seen in 57.7% (15/26) of oocytes. This decrease in spindle tapering at both ends of the spindle may be due to the abnormal DNA projections rather than direct defects in spindle assembly caused by decreased Top2 levels.

The ability of *Top2* RNAi/*matαGAL* oocytes to organize a bipolar spindle is further illustrated by live imaging. Video S1 shows a bipolar spindle quickly forming after germinal vesicle breakdown in a *Top2* RNAi/*matαGAL* oocyte. After the completion of spindle assembly, the spindle remained bipolar and the achiasmate chromosomes remained associated with the autosomes for the duration of imaging. In 11 time-lapses of *Top2* RNAi*/matαGAL* oocytes undergoing germinal vesicle breakdown, eight successfully formed a bipolar spindle. Additionally, all nine oocytes that had already completed spindle assembly by the start of live imaging maintained bipolar spindles for the duration of imaging. These results argue strongly that defects in spindle assembly are not the primary cause of the centromere-led abnormal projections in *Top2* RNAi*/matαGAL* oocytes.

### DNA projections observed in *Top2* RNAi*/matαGAL* oocytes are composed of heterochromatin

**Figure 3:**
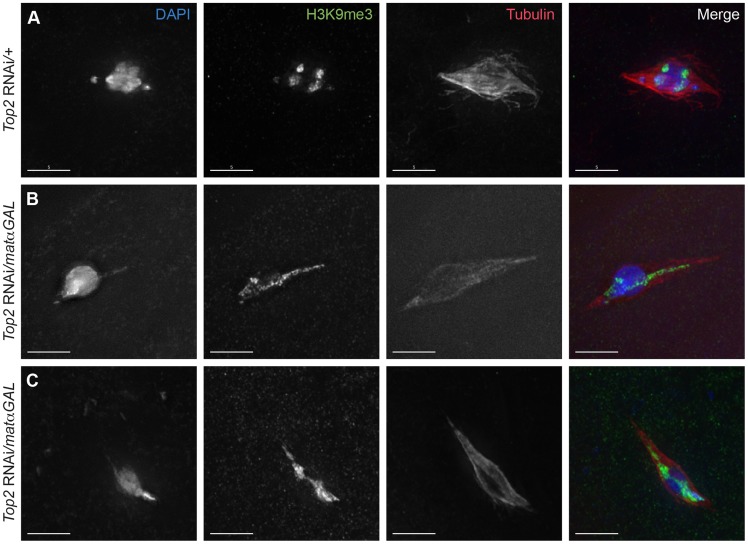
Aberrant DNA projections are composed of heterochromatin. DNA is labeled with DAPI (blue), heterochromatin is labeled with an antibody recognizing histone 3 trimethylated on lysine 9 (H3K9me3) (green), and the spindle is labeled with an antibody recognizing α-tubulin (red). (A) *Top2* RNAi*/+* control oocyte with achiasmate *4^th^* chromosomes that have moved towards the spindle poles. The *4^th^* chromosomes and the centromeric regions of the chromosomes are labeled with H3K9me3 and are oriented towards opposite spindle poles. (B–C) *Top2* RNAi*/matαGAL* oocytes containing abnormal DNA projections that are labeled with the H3K9me3 antibody. Additionally, the H3K9me3 antibody localization in the chromosome mass of both oocytes is not oriented towards opposite spindle poles. Images are projections of partial Z-stacks. Scale bars are 5 microns.

The regions of DNA that stain brightest with DAPI are typically the heterochromatic regions, such as those near the centromeres and a large portion of the *4^th^* chromosomes. At metaphase I, the DAPI-bright DNA regions are oriented towards opposite spindle poles and are located at the ends of the chromosome mass [7]. In *Top2* RNAi*/matαGAL* oocytes, DAPI-bright regions could be observed in aberrant configurations. For example, in Figure 2B, a single DAPI-bright region is present in the middle of the chromosome mass, and in Figure 2C a single DAPI-bright region is present at one end of the chromosome mass. To investigate this further, we used an antibody recognizing histone 3 trimethylated on lysine 9 (H3K9me3), a chromatin modification associated with heterochromatin [25], [26]. While in *Top2* RNAi*/+* oocytes the H3K9me3 signals were observed oriented towards both spindle poles, in *Top2* RNAi*/matαGAL* the H3K9me3 signal was present on the DNA within the projections (Figure 3). H3K9me3-positive regions could also be seen splayed across the chromosome mass (Figure 3). These results suggest that the projections are composed of heterochromatic sequences, and we will show below that knockdown of *Top2* causes a failure of heterochromatic regions to properly orient.

### Heterochromatic regions fail to separate properly in *Top2* RNAi-expressing oocytes

As the DNA projections appeared to be composed of heterochromatin, as based on the H3K9me3 antibody, we next wanted to determine whether these projections were simply caused by the failure of the achiasmate *4^th^* chromosomes to move fully toward the spindle poles. Because heterochromatic threads connect achiasmate chromosomes during prometaphase I, we also wanted to know how heterochromatic regions would be affected by decreased Top2 levels. Finally, we wanted to determine whether homologous chromosome orientation would be affected by *Top2* RNAi expression.

**Figure 4:**
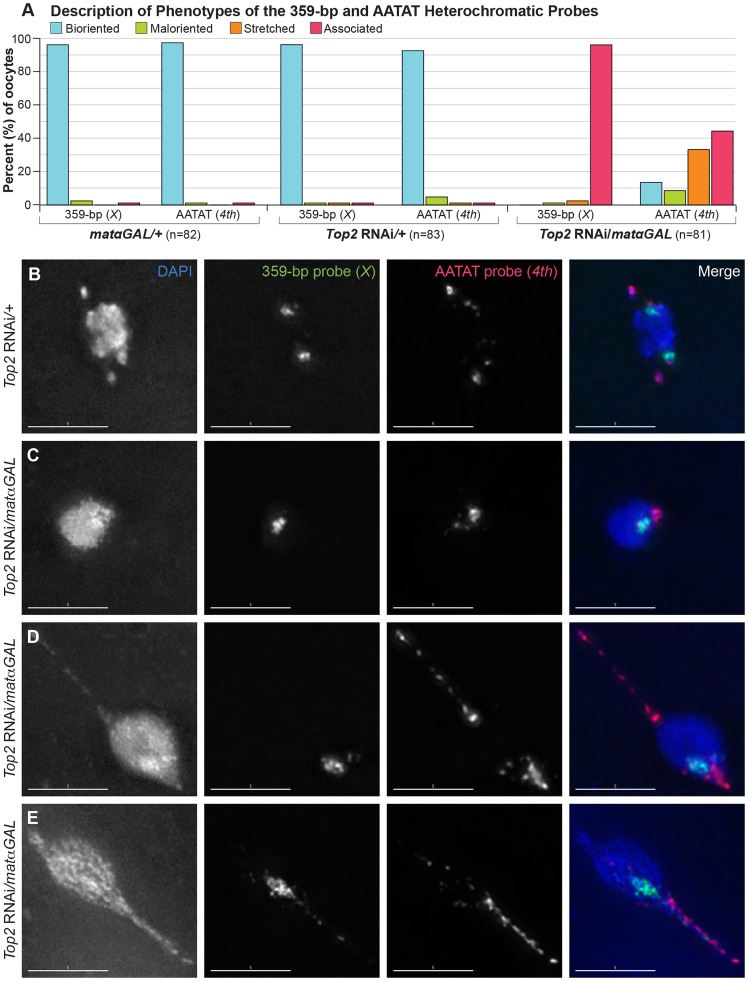
*Top2* RNAi*/matαGAL* oocytes show defects in chromosome biorientation and in the separation of heterochromatic regions of the *X* and *4^th^* chromosomes. (A) Quantification of the phenotypes observed for the 359-bp and AATAT heterochromatic probes. Bioriented represents oocytes with two FISH probe foci oriented in opposite directions. Maloriented represents two FISH probe foci oriented in the same direction. Stretched represents FISH signal that is highly elongated rather than in discrete foci. Associated represents oocytes with only a single FISH probe focus. (B–E) DNA is labeled with DAPI (blue), a FISH probe targeting the 359-bp repeat predominantly on the *X* chromosome and a minor region on the *3^rd^* chromosome is in green, and a FISH probe to the AATAT heterochromatic repeat on the *4^th^* chromosome and a minor repeat on the *X* chromosome is shown in red. (B) *Top2* RNAi*/+* control oocyte with two foci for both probes oriented in opposite directions, indicating proper biorientation of the *X* and *4^th^* chromosomes. (C–E) Shown are examples of *Top2* RNAi*/matαGAL* oocytes displaying aberrant separation of heterochromatic regions. (C) Probes indicate that both heterochromatic regions have failed to separate. (D) The *X* chromosomes have failed to separate. The *4^th^* chromosomes have separated and bioriented, but one *4^th^* chromosome appears to be highly stretched out and present in an abnormal DNA projection. (E) Both heterochromatic regions have failed to separate, but the AATAT repeat and, to a small degree, the 359-bp region have stretched into the abnormal DNA projection. For the 359-bp probe 96.3% (79/82) of *Top2* RNAi*/matαGAL* oocytes displayed a complete failure of the probe to separate. In two (2.4%) *Top2* RNAi*/matαGAL* oocytes the 359-bp probe appeared stretched out and one oocyte (1.2%) had 2 maloriented foci. Images are projections of partial Z-stacks. Scale bars are 5 microns.

We utilized fluorescent *in situ* hybridization (FISH) probes recognizing heterochromatic regions of each chromosome to look at the separation of specific heterochromatic regions and to assess biorientation of homologs in females bearing structurally normal *X*, *2^nd^*, *3^rd^*, and *4^th^* chromosomes. We first examined a probe recognizing the 359-bp satellite, which is primarily localized to the *X* chromosome, with a minor region on the *3^rd^* chromosome [27]. In prometaphase I and metaphase I control *Top2* RNAi*/+* and *matαGAL/+* oocytes, two large, well-separated and bioriented fluorescent 359-bp signals were observed in 96.4% (80/83) and 96.3% (79/82) of oocytes, respectively (Figure 4A, B). Furthermore, in immunoFISH studies using the 359-bp probe and an α-tubulin antibody to mark the spindle, the *X* chromosomes were properly oriented toward opposite poles of the bipolar spindle in 100% (16/16) of *Top2* RNAi/+ oocytes (Figure S2A).

However, when we followed the *X* chromosomal heterochromatin using the 359-bp probe in *Top2* RNAi/*matαGAL* oocytes, none of the oocytes (0/81) displayed two bioriented 359-bp foci. Instead, the 359-bp heterochromatic region failed to fully separate in 96.3% (78/81) of *Top2* RNAi/*matαGAL* oocytes that should have been in prometaphase I or metaphase I, based on egg chamber stage (Figure 4A, C–E). In an immunoFISH experiment with α-tubulin, the lack of separation of the 359-bp heterochromatic region could be observed even on fully formed bipolar spindles in *Top2* RNAi/*matαGAL* oocytes. Two bioriented 359-bp foci were not observed in any of the 16 oocytes examined, which is to say that the 359-bp regions failed to separate for 100% (16/16) of the spindles. This result indicates that the failure to separate heterochromatic regions is not likely to be due to a defect in meiotic progression or spindle assembly after germinal vesicle breakdown (Figure S2B, C).

We then asked whether the heterochromatin of the achiasmate *4^th^* chromosomes would fail to separate in *Top2* RNAi/*matαGAL* oocytes. A FISH probe recognizing the AATAT repeat present throughout the *4^th^* chromosomes was examined. Two large, well-separated and bioriented AATAT probe foci were observed in 92.8% (77/83) of *Top2* RNAi*/+* control oocytes (Figure 4A, B) and in 97.6% (80/82) of *matαGAL/+* oocytes (Figure 4A). However, separation of the AATAT *4^th^* chromosome probe was strongly impaired in *Top2* RNAi/*matαGAL* oocytes. The *4^th^* probe failed to separate in 44.4% (36/81) of *Top2* RNAi/*matαGAL* oocytes (Figure 4A, C). In 33.3% (27/81) of *Top2* RNAi/*matαGAL* oocytes, the *4^th^* probe signal was highly elongated, often extending into one or more projections (Figure 4A, D, E). This phenotype suggests that the AATAT repeat of the *4^th^* chromosomes has become stretched out and indicates that at least some of the projections are composed of *4^th^* chromosome heterochromatin. In 8.6% (7/81) of oocytes, two foci could be distinguished, but the foci were still oriented toward the same side of the DNA mass, indicating a failure in *4^th^* chromosome biorientation. However, 13.6% (11/81) of oocytes did display two separated and bioriented foci, indicating that at least in some cases this region of the *4^th^* chromosome can successfully separate and biorient in *Top2* RNAi/*matαGAL* oocytes. These data demonstrate that decreased Top2 levels result in defects in *4^th^* chromosome biorientation and the full separation of the AATAT heterochromatin repeat.

**Figure 5:**
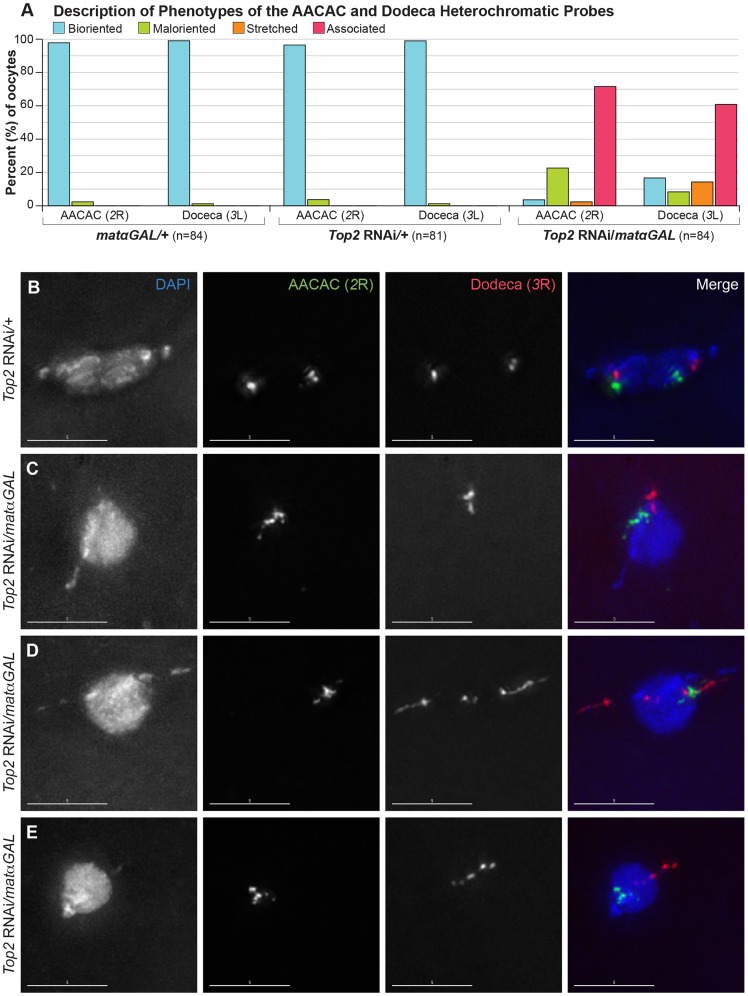
*Top2* RNAi*/matαGAL* oocytes show defects in chromosome biorientation and in the separation of heterochromatic regions of the *2^nd^* and *3^rd^* chromosomes. (A) Quantification of the phenotypes observed for the AACAC and Dodeca heterochromatic probes. Bioriented represents oocytes with two FISH probe foci oriented in opposite directions. Maloriented represents two FISH probe foci oriented in the same direction. Stretched represents FISH signal that is highly elongated rather than discreet foci. Associated represents oocytes with only a single FISH probe focus. (B–E) DNA is labeled with DAPI (blue), a FISH probe targeting the AACAC repeat on the right arm of the *2^nd^* chromosome is in green, and a FISH probe to the Dodeca heterochromatic repeat on the right arm of the *3^rd^* chromosome is shown in red. (B) *Top2* RNAi*/+* control oocyte with two foci for both probes oriented in opposite directions, indicating proper biorientation of the *2^nd^* and *3^rd^* chromosomes. (C–E) Examples of *Top2* RNAi*/matαGAL* oocytes displaying aberrant separation of heterochromatic regions. (C) Probes indicate that both heterochromatic regions have failed to separate. (D) The AACAC heterochromatic region of the *2^nd^* chromosomes has failed to separate. The Dodeca heterochromatic region of the *3^rd^* chromosome has separated but the heterochromatic region is highly stretched out and present in the abnormal DNA projections. (E) Both heterochromatic regions appear abnormal and the *2^nd^* and *3^rd^* chromosomes are improperly oriented so as to segregate away from each other. Images are projections of partial Z-stacks. Scale bars are 5 microns.

Heterochromatin threads have been observed connecting the *4^th^* and *X* homologs during prometaphase I in *Drosophila* oocytes [6]. The presence of these threads may make heterochromatic regions of these chromosomes more sensitive to decreased Top2 levels. To determine whether a heterochromatic region of the chiasmate *2^nd^* chromosomes would also be affected by decreased Top2 levels, we examined a heterochromatic probe recognizing the AACAC repeat on the right arm of the *2^nd^* chromosome in prometaphase I and metaphase I oocytes [27]. Two bioriented AACAC foci were present in 96.3% (78/81) of *Top2* RNAi/+ oocytes and 97.6% (82/84) of *matαGAL/+* oocytes (Figure 5A, B). In contrast, *Top2* RNAi/*matαGAL* oocytes displayed only a single focus for the AACAC probe in 71.4% (60/84) of oocytes that had likely completed spindle assembly based on egg chamber stage, indicating that, like heterochromatic regions on the *X* and *4^th^* chromosomes, this heterochromatic region of *2R* is also defective in its ability to separate (Figure 5A, C, D, E). In 22.6% (19/84) of oocytes, two maloriented AACAC probe foci were observed. The probe was stretched in 2.4% (2/84) of *Top2* RNAi/*matαGAL* oocytes and two bioriented foci were observed in only 3.6% (3/84) of oocytes. These results indicate that a decreased Top2 level affects the separation of the AACAC heterochromatic region and the proper biorientation of the *2^nd^* chromosomes during meiosis I.

Finally, we asked whether a heterochromatic region of the chiasmate *3^rd^* chromosomes would show similar defects. We utilized a FISH probe to the heterochromatic Dodeca satellite on the *3^rd^* chromosomes. Two bioriented Dodeca foci were present in 98.8% (80/81) of *Top2* RNAi/+ control oocytes and 98.8% (83/84) of *matαGAL/+* control oocytes during prometaphase I and metaphase I (Figure 5A, B). Upon *Top2* RNAi expression, the Dodeca heterochromatic repeat failed to separate in 60.7% (51/84) of oocytes (Figure 5A, C). In 13.1% (11/84) of *Top2* RNAi/*matαGAL* oocytes, the Dodeca FISH signals were highly stretched, often extending into a projection (Figure 5A, D, E). This observation demonstrates that the projections can be composed of chiasmate chromosome heterochromatin as well as that of the achiasmate *4^th^* chromosomes. The Dodeca probe was bioriented in 16.7% (14/84) of oocytes, while two foci were maloriented in 8.3% (7/84) of oocytes. This illustrates that *3^rd^* chromosome heterochromatin is also affected by decreased *Top2* expression. We also observed that in 36.4% (30/83) of *Top2* RNAi/*matαGAL* oocytes the *2^nd^* and *3^rd^* chromosomes segregated away from each other (Figure 5E) and that in 24.1% (20/83) of oocytes, both sets of *2^nd^* and *3^rd^* chromosomes were oriented in the same direction. These results once again illustrate that Top2 is involved in the proper biorientation of chiasmate chromosomes during meiosis I.

In conclusion, heterochromatic regions on all four chromosomes showed defects in their ability to fully separate at prometaphase I and metaphase I in *Top2* RNAi/*matαGAL* oocytes. Additionally, these chromosomes displayed a failure to properly biorient. These data support the idea that Top2 is involved in releasing the bonds that hold heterochromatic regions together during prophase [5]. Even if heterochromatic regions could become fully separated at anaphase I in *Top2* RNAi/*matαGAL* oocytes, the orientation of homologous chromosomes toward the same pole would lead to high levels of chiasmate and achiasmate chromosome missegregation [7], [28]. Although the primary conclusion to be drawn here is that Top2 is required to separate heterochromatic regions on all four chromosomes, perhaps the more interesting inference is that the lack of heterochromatic separation argues strongly for heterochromatic entanglements that affect all four heterochromatic regions tested.

### A mutated RNAi construct fails to cause defects in the separation of heterochromatic regions

To ensure that the defects in heterochromatic separation observed with the *Top2* RNAi construct were caused specifically by the 21-nucleotide sequence targeting *Top2*, we constructed a mutated RNAi construct and tested its effects on spindle assembly and heterochromatin separation (see Materials and Methods). The *Top2* RNAi construct shares homology with a second locus (*CG33296*) at 18 of the 21 nucleotides. Rather than randomly mutagenizing or scrambling the original construct, the Top2 RNAi construct was mutated to match the *CG33296* gene, even though a meiotic function has not been speculated for this gene product nor has it been reported to show ovarian expression (FlyBase). We hoped the targeted mutagenesis of the *Top2* RNAi construct would provide a better control for the specificity of the construct compared to a randomly scrambled construct that would only address the potential general effects of RNAi induction in the ovary.

Bipolar spindles could be observed in oocytes from *CG33296* RNAi/*matαGAL* mothers (Figure S3A) and at least one *4^th^* chromosome was separated from the autosomes in 27.6% (8/29) of oocytes. FISH experiments of *CG33296* RNAi/*matαGAL* oocytes showed that all four heterochromatic FISH probes were well separated and properly bioriented in the majority of oocytes (97.8% [44/45] 359 bp, 96.7% [29/30] AACAC, 100% [30/30] Dodeca, and 97.8% [44/45] AATAT) (Figure S3). These data support the conclusion that the effects of the *Top2* RNAi construct are due to decreased Top2 levels rather than off-target RNAi effects.

### Euchromatic regions can separate during mid-prophase in *Top2* RNAi*/matαGAL* oocytes

Dernburg *et al.* [5] noted that *Drosophila* oocytes undergo a modified diplotene phase, in which euchromatic regions appear to separate as early as stage 3–4, while heterochromatic regions remain tightly paired until prometaphase I. Thus, we did not expect that the RNAi knockdown generated in *Top2* RNAi*/matαGAL* oocytes performed in these studies would impair separation of euchromatic regions. To verify this hypothesis, we examined mid-prophase oocyte nuclei to determine whether euchromatic regions could separate in *Top2* RNAi*/matαGAL* oocytes. Dernburg *et al.* [5] observed separation of the euchromatic histone locus in 54.5% (36/66) of mid-prophase oocytes. Similarly, we observed that *Top2* RNAi*/matαGAL* oocytes exhibited two foci of fluorescence for a BAC probe to polytene band *3C* of the *X* chromosome in 51.1% (23/43) of prophase oocytes compared to 48.9% (22/45) of *Top2* RNAi*/+* control oocytes (Figure S4). Using a BAC probe to polytene bands *7DE* of the same chromosome, 57.6% (19/33) of *Top2* RNAi/*matαGAL* oocytes were observed to have two foci compared to 47.9% (23/48) of *Top2* RNAi*/+* control oocytes (Figure S4). These results suggest that while Top2 is required for the separation of heterochromatic regions following nuclear envelope breakdown, Top2 is either not required for the separation of euchromatic regions during mid-prophase, or the *matαGAL* driver does not knock down the level of Top2 enough to affect separation of euchromatic regions. However, these results do not rule out the possibility that there are earlier euchromatic entanglements that are formed during replication and then resolved prior to *Top2* RNAi induction. Our results are consistent with our view that DNA entanglements that persist into mid-to-late prophase, as revealed by Top2 knockdown, are specific to the heterochromatin.

### Embryos from *Top2* RNAi-expressing mothers fail to initiate proper mitotic divisions

**Figure 6:**
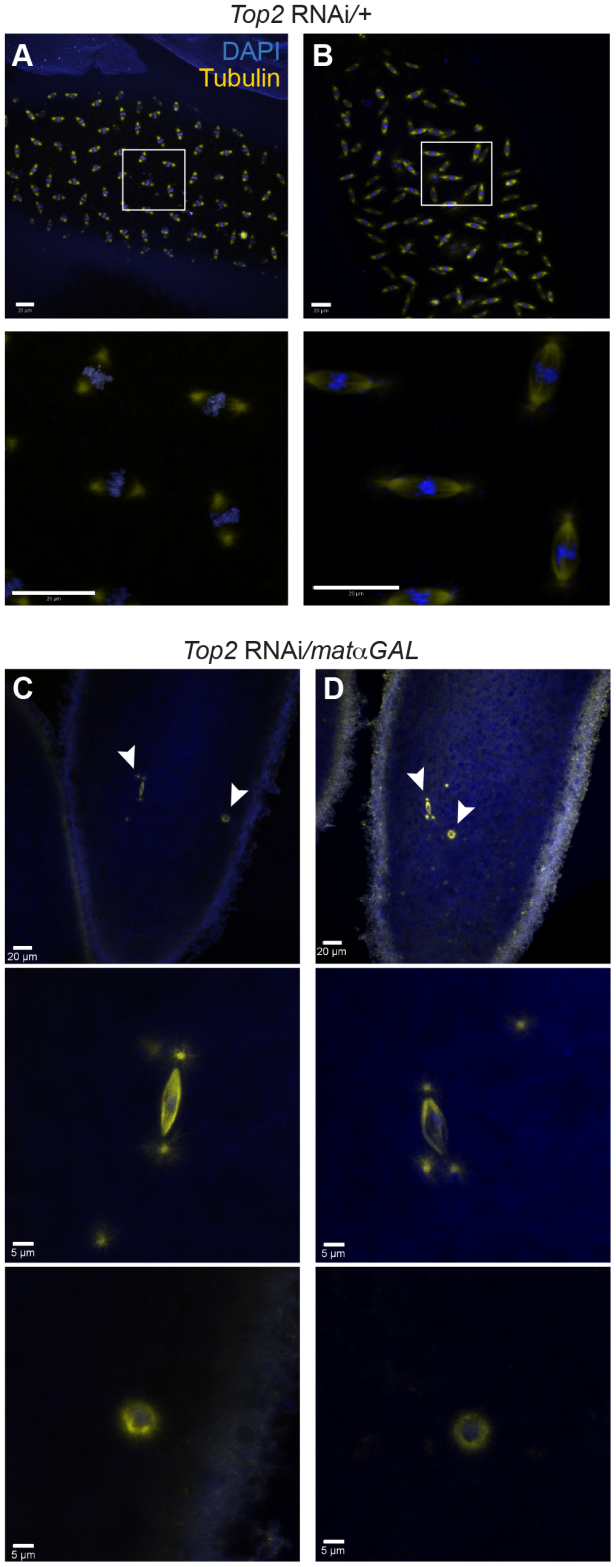
Embryos from *Top2* RNAi*/matαGAL* mothers appear to be arrested in meiosis I. DNA is labeled with DAPI (blue) and spindles are labeled with an antibody recognizing α-tubulin (yellow). (A and B) *Top2* RNAi*/+* embryos displaying normal development. Boxed regions are shown magnified below each image. (C and D) Embryos from *Top2* RNAi/*matαGAL* mothers with two nuclei indicating proper fertilization but an arrest in meiosis I. 17/20 embryos from *Top2* RNAi*/matαGAL* mothers contained only 2 nuclei. Of the remaining 3 embryos, one contained only a single nucleus and 2 appeared to undergo an aberrant division. Arrowheads indicate regions magnified below both images. Images are projections of partial Z-stacks. Scale bars are 20 microns.

A defect in the ability to properly separate heterochromatic regions during prometaphase and metaphase of meiosis I would likely result in a failure to properly complete meiosis and enter into the first mitotic divisions after fertilization. In embryos from *Top2* RNAi/+ control mothers, we observed normal embryonic development in 30/30 (100%) embryos, indicating that meiosis was successfully completed (Figure 6A). In contrast, 0.0% (0/20) of embryos from *Top2* RNAi*/matαGAL* mothers initiated proper embryonic development, with 85.0% (17/20) containing only two nuclei: a small nucleus with a centriolar spindle that was presumed to be the paternal pronucleus and a larger, round nucleus (Figure 6B, C). This round nucleus was surrounded by α-tubulin that was not organized into a bipolar shape. Because this nucleus did not resemble the typical rosette structure of embryos, it was presumed to be the oocyte nucleus that had failed to exit meiosis I. The three exceptions are described in the legend of Figure 6. This absence of mitotic entry led to a complete failure of embryos from *Top2* RNAi-expressing mothers to hatch (0.0% [0/447]).

## Discussion

We have shown that knockdown of *Top2* during prophase of meiosis I in *Drosophila* oocytes results in defects in homolog segregation and sterility. Heterochromatic regions of all four chromosomes failed to properly separate, leading to a failure of chromosomes to properly biorient during meiosis I. Additionally, achiasmate chromosomes showed defects in their ability to move away from the autosomes towards the spindle poles in prometaphase I. Instead, abnormal chromosomal projections were present. These DNA projections displayed several differences compared to the heterochromatic threads observed in wild-type prometaphase I oocytes. Specifically, the projections did not appear to directly connect two chromosomes and, more importantly, contained centromeres, which are not present in wild-type DNA threads. These attributes suggest that chromosomes initiate centromere-led movement but are anchored by DNA entanglements at the center of the spindle, resulting in the stretching out of chromosomal regions.

One might imagine that one component of these defects reflects a role of Top2 in resolving chiasmata. Indeed, in yeast, conditional mutants of *top2* caused a meiotic cell cycle arrest that was partially alleviated by simultaneously eliminating recombination, indicating that in yeast, some of the targets of Top2 during meiosis are recombination dependent [17], [18]. Several lines of evidence suggest that this is not the case in *Drosophila*. First, the *matαGAL* driver used in the *Drosophila* oocytes does not appear to be strongly expressed until after recombination is thought to be finished [23]. Second, recombination is suppressed in heterochromatic regions near the centromeres [29]. Therefore, it seems more likely that the defects in heterochromatic separation during *Top2* knockdown are due to the failure to resolve the heterochromatic threads observed during prometaphase I rather than a failure of Top2 to resolve DNA entanglements during the repair of double-strand breaks initiated in early prophase. Third, the AATAT heterochromatic repeat of the *4^th^* chromosomes also shows defects in separation. Since the *4^th^* chromosomes, which never undergo crossing over and are thus obligately achiasmate, also fail to properly separate heterochromatic regions [1], the defects in *4^th^* chromosome heterochromatin separation cannot be due to homologous connections formed as a result of recombination. Finally, the heterochromatic threads observed during prometaphase I in *Drosophila* oocytes are not dependent on recombination, since they are observed in mutants of the *Drosophila spo11* homolog, *mei-W68* (Figure S5). While Top2 appears to affect the heterochromatic regions by resolving DNA entanglements that are recombination independent, it is unknown whether or not Top2 plays a role in the resolution of chiasmata at anaphase I. Since knockdown of Top2 results in metaphase I arrest, a role at anaphase I in chiasmata resolution cannot be assessed under these conditions.

The observation that *4^th^* chromosome sequences are present within the DNA projections in *Drosophila* oocytes is not surprising given that the *4^th^* chromosomes often move precociously towards the spindle poles during prometaphase I. However, it is more difficult to explain the stretching of *3^rd^* chromosome heterochromatic sequences into some projections and the multiple CID foci within the projections. These results may indicate that the centromeres of the chiasmate chromosomes are also attempting to move towards the poles in *Top2* RNAi-expressing oocytes. One possibility is that these projections are the consequence of a failed attempt by the oocyte to separate the heterochromatic regions. The four heterochromatic regions examined varied in the extent that they failed to separate. These differences may be due to a difference in the number of DNA entanglements between homologous heterochromatic regions in the oocytes.

The 359-bp region displayed the highest failure to separate upon *Top2* knockdown. Several lines of evidence have suggested that the 359-bp heterochromatic region of the *X* may be handled differently by the cell than other heterochromatic regions. First, Ferree and Barbash [30] demonstrated that the hybrid lethality between *D. simulans* females and *D. melanogaster* males is due to the formation of anaphase bridges containing the 359-bp repeat region during mitosis in hybrid embryos. The authors speculate that *D. simulans* may lack factors for proper condensation of the 359-bp region. More recently, Ferree *et al.* [31] demonstrated that the lethality caused by some circularized *X-Y* ring chromosomes is also due to anaphase bridging of the 359-bp repeat in embryos. In both instances, Top2 localized to the anaphase bridges. Additionally, during the mitotic divisions of the *Drosophila* germarium, the 359-bp region is highly paired while the AACAC and Dodeca regions of the *2^nd^* and *3^rd^* chromosomes are mostly unpaired [32]. These results, as well as the complete failure of the 359-bp region of the *X* to separate in over 90% of oocytes when *Top2* is knocked down, suggest that the 359-bp region may be especially prone to form DNA entanglements during replication (both between homologs and sisters) or that these entanglements may be processed differently than those in other heterochromatic regions. Additionally, *in vivo* studies of Top2 cleavage sites during mitosis in *Drosophila* showed a major cleavage site in the 359-bp repeat [33]. A failure to cleave this site when Top2 levels are decreased in meiosis I may contribute to the high failure of the 359-bp repeat to separate in *Top2* RNAi oocytes.

### Comparing the meiotic effects of Top2 knockdown in males versus females

In a parallel study examining decreased Top2 levels during *Drosophila* male meiosis, in which recombination does not occur [34], Mengoli *et al.* (cosubmitted) observed phenotypes similar to those seen by us in oocytes. Homologs, as well as sister chromatids, frequently failed to separate during meiosis I and meiosis II in *Drosophila* males, despite the formation of bipolar spindles similar to *Drosophila* oocytes. Homologs were stretched out into anaphase bridges at meiosis I, a phenotype which has similarities to the stretched out chromosomal projections in oocytes. These results indicate that Topoisomerase II may resolve similar DNA connections in both sperm and oocytes. Thus, the cells are responding in a similar fashion to deal with the failure of the resolution of these homologous connections. It is worth noting, however, that Mengoli *et al.* (cosubmitted) observed defects in euchromatic regions as well as heterochromatic regions of the chromosomes during male meiosis, while in oocytes, only heterochromatic regions were affected. This difference likely reflects the fact that Mengoli *et al.* (cosubmitted) examined mutations affecting Top2 levels at the start of meiosis while in *Drosophila* females the Top2 RNAi construct is not expressed until mid-late prophase.

### Comparing the meiotic and mitotic roles of Top2

Decreasing the level of Top2 in mitotic cell types causes phenotypes that are both similar to and divergent from those phenotypes observed during *Drosophila* female and male meiosis (Mengoli *et al.* cosubmitted). *Top2* RNAi in mitotic *Drosophila* S2 cells led to the formation of DNA projections [35]. Although these chromosomal projections appeared similar to the ones in oocytes, CID foci were not observed within the DNA projections in S2 cells, while one or more CID foci were present in the projections in oocytes. This suggests that while the projections in oocytes are at least partially centromere led, the S2 projections are composed of the arms of the chromosomes. *Top2* RNAi expression in S2 cells results in extensive chromosome lagging, chromosome bridging during anaphase, and chromosome missegregation [36]. Oocytes expressing *Top2* RNAi seem to arrest in metaphase I before chromosome bridging can manifest, but chromosome missegregation is evident in oocytes as well.

We should also note that knockdown of Top2 in Kc cells leads to a decrease in euchromatic pairing without affecting the pairing of heterochromatic regions [37], [38]. However, evidence suggests Top2 is acting in different ways in each system. In meiosis, heterochromatic regions remain associated at higher levels than euchromatic regions during mid to late prophase [5], while euchromatic pairings persist longer than heterochromatic pairings during mitosis in cultured cells [37], [38]. Our data suggest that the lack of heterochromatin dissociation is due to the failure of Top2 to resolve DNA entanglements within the heterochromatin, while there is no evidence for the persistence of similar entanglements between euchromatic regions upon knockdown of Top2 in cell culture.

The study by Mengoli *et al.* (cosubmitted) suggests that different phenotypes manifest in *Drosophila* larval neuroblasts depending on the residual level of Top2. Additionally, different cell types, for example sperm, were more sensitive to decreases in Top2 levels. This study, as well as others in *Drosophila* [21], [39], illustrates the complexity of understanding the function of Top2 in resolving various types of DNA entanglements. For example, expression of the *Top2* RNAi construct using the nanos-Gal4:VP16 driver, which is expressed beginning in germline stem cells [40], led to minimal ovarian development (Figure S6), suggesting that high levels of Top2 expression are likely necessary to resolve DNA entanglements caused by replication in the germline stem cell divisions and/or the cystoblast divisions. This hinders the examination of the role of Top2 in such processes as replication, recombination, and chromosome condensation early in oogenesis.

Knocking down topoisomerase II enzymes using RNAi or chemically inhibiting its two isoforms in mitotic human cell lines leads to a number of defects, including entangled chromosomes, chromosome segregation defects, cell cycle delays, and in some cases cell cycle arrest [41]–[43]. Most interesting is that chemically inhibiting Topoisomerase IIα in HeLa cells has been reported to increase the number and duration of PICH (Plk1-interacting checkpoint helicase)-positive ultrafine DNA bridges that connect centromeres during anaphase of mitosis, including to non-centromeric regions of the chromosomes. These results indicate that Topoisomerase II enzymes resolve DNA entanglements prior to anaphase in addition to those observed at the centromeres in mitotic cells [44], [45]. These PICH-positive ultrafine bridges have several similarities to the DNA threads observed during prometaphase I of *Drosophila* oocytes, in that they are composed of heterochromatin and connect segregating chromosomes. While mitotic DNA entanglements are between sister chromatids and some of the meiotic entanglements are likely between homologs, the results suggest that Topoisomerase II enzymes may play a conserved role in resolving chromosomal entanglements in mitosis and meiosis.

Determining the mechanism by which Topoisomerase II functions to resolve mitotic entanglements may provide insight into its potential role in resolving the meiotic threads observed by Hughes *et al*. [6]. In HeLa cells, centromeric cohesion appears to protect centromeric DNA threads from resolution until anaphase I when this cohesion is lost, and a similar mechanism is believed to protect centromeric concatenations at centromeres until anaphase II during the mouse male meiotic divisions [46], [47].

Based on these studies, it is at least possible that in *Drosophila* oocytes entanglements may form during replication in both heterochromatic and euchromatic regions, but euchromatic entanglements may be more accessible to resolution by Top2 immediately after replication. These entanglements would be resolved before the *Top2* RNAi construct is induced. Heterochromatic entanglements may be protected from early resolution due their conformation after replication or the presence of heterochromatin binding proteins. Top2 would be unable to resolve these entanglements until the karyosome reorganizes for prometaphase I or until tension is provided on the DNA by microtubules, as has been proposed for the resolution of entanglements by Top2 in yeast [48]. At this stage Top2 levels would be reduced in *Top2* RNAi-expressing oocytes, leading to a failure to resolve these entanglements. Alternatively, heterochromatic regions may be particularly prone to forming entanglements due to the repetitive nature of heterochromatic DNA, and thus more sensitive to decreased Top2 levels.

### Does Top2 knockdown also affect chromosome condensation in late-stage *Drosophila* oocytes?

Topoisomerase II enzymes have also been implicated in regulating chromosome condensation in a number of cell types and organisms [12]. For example, Mengoli *et al.* (cosubmitted) reported that the centric heterochromatin appeared undercondensed in some neuroblasts from Top2 RNAi-expressing larvae. Additionally, strong knockdown of *Top2* levels in *Drosophila* S2 cells led to a large and quantitative change in chromosome condensation [35].

These observations led us to ask whether some of the phenotypes observed in this study might be the consequence of the effect of Top2 depletion on chromosome condensation, especially in the pericentric heterochromatin. Global chromosome condensation in *Top2* RNAi-expressing oocytes looked similar to wild-type oocytes, but small changes in chromosome condensation would be obscured by the close proximity of the chromosomes at prometaphase I and the high level of condensation of the chromosomes. It is thus possible that chromosomes are undergoing mild decreases in condensation, particularly in heterochromatic regions, when Top2 levels are decreased. Decreased condensation could contribute to the stretched out phenotype observed with the *3^rd^* and *4^th^* chromosome FISH probes and to the centromere-led chromosomal projections. However, the effects on condensation that we observe appear to be too weak to account for the entanglements and stretching that we observe.

### A model for the meiotic role of Top2

These observations lead to a speculative model for the cause of the defects in *Top2* RNAi-expressing oocytes. In wild-type oocytes, DNA entanglements form between the highly repetitive DNA sequences of heterochromatic regions. These entanglements could form during replication by stalled replications forks that can occur in the repetitive heterochromatic regions or by intertwinings that could form as chromosomes are replicated in close proximity. Entanglements within the heterochromatic regions would not be immediately resolved. Therefore, these entanglements could help hold heterochromatic regions of homologous chromosomes tightly together during prophase, including those chromosomes that, like the *4^th^* chromosomes, fail to undergo recombination [5]. As germinal vesicle breakdown approaches, many of these entanglements would have to be resolved by Top2 in order for chromosomes to separate and biorient properly during prometaphase I and metaphase I, possibly assisted by karyosome reorganization and/or microtubule attachments to the chromosomes. The chromatin threads observed during prometaphase I could be the entanglements that failed to be resolved during late prophase or those protected from resolution to facilitate the biorientation of achiasmate chromosomes. Top2 and/or other enzymes would then resolve these final DNA threads by anaphase I. In oocytes with decreased levels of Top2, many of the DNA entanglements would not be resolved during meiosis I, leading to a failure of homologous heterochromatic regions to separate. In some cases, heterochromatic regions appear to attempt separation, but the DNA entanglements hold the chromosomes together at one or more places and the rest of the heterochromatic regions of the chromosomes become highly stretched out. The centromere-led DNA projections apparently occur when chromosomes attempt separation despite the existence of heterochromatic entanglements. Since the heterochromatic regions would still be locked together at egg activation when meiosis resumes, chromosomes would be unable to segregate to opposite spindle poles at anaphase I and ultimately, the oocytes would fail to exit meiosis I. Our results indicate that Top2 plays an important role in resolving homologous DNA entanglements in *Drosophila* oocytes. These results also suggest that the formation of such entanglements (by whatever mechanism) may be a characteristic of the meiotic process.

## Materials and Methods

### Stocks

Flies were maintained on standard food at 25°C. Fly stocks used for RNAi experiments were *y w; spa^pol^*, *w; {matα4-GAL-VP16}V37* (Bloomington 7063), *y^1^ sc^1^ v^1^; P{y[+t7.7] v[+t1.8] = TRiP. GL00338}attP2* (Bloomington 35416) an RNAi construct targeting the *Top2* (*CG10223*) gene, and y v; CG33296 RNAi (described below). To obtain control flies containing only one copy of the driver or RNAi construct, the designated stocks were crossed to *y w; spa^pol^* flies. Transheterozygotes mutant for *mei-W68* (*CG7753*) were made from the following stocks: *y/B^S^ Y; mei-W68^Z1049^ cn bw/SM6a* and *y/y^+^ Y; mei-W68^Z4572^ cn bw/Cyo* [49]. The genotype of the nanos-Gal4:VP16 driver flies was *y w/y^+^ Y; nanos-Gal4:VP16; spa^pol^*.

### Immunostaining

Immunostaining of late stage oocytes was carried out as described [8] under conditions to limit activation. For experiments enriching for prometaphase I oocytes, females were yeasted for 2–3 days with males. For preparations enriching for metaphase I oocytes, virgin females were yeasted for 4–5 days post-eclosion [7]. Ovaries were treated with the primary and secondary antibodies described below and with 1.0 µg/mL 4′6-diamididino-2-phenylindole (DAPI) to label the DNA and mounted in ProLong Gold (Invitrogen). Immunostaining of embryos was carried out as described [50]. The DNA was labeled with 2.5 µg/mL Hoechst 34580 (Invitrogen) and mounted in ProLong Gold (Invitrogen).

Primary antibodies were used at the following concentrations: rat anti-α-tubulin (AbD Serotec, NC 1∶250), mouse anti-α-tubulin DM1a (Sigma-Aldrich 1∶100), rat anti-CID [51] 1∶1000), rabbit anti-trimethylated-histone-3 at lysine 9 (AbCam 1∶250) and rabbit anti-phosphorylated-histone 3 at serine 10 (Millipore 3∶1000). Secondary Alexa-488, Alexa-555, or Alexa-647 conjugated antibodies (Molecular Probes) were used at a dilution of 1∶400.

### Fluorescent in-situ hybridization

FISH was carried out as described [52], with the following modifications. Alexa Fluor 488 was conjugated to a region of the 359-bp repeat on the *X* chromosome, and the AATAT repeat on the *4^th^* chromosome was conjugated to Cy3 [5], [30]. Both probes were denatured at 91°C and hybridization was carried out at 31°C. For the Alexa Fluor 488-labeled AACAC probe on the right arm of chromosome *2* and the Alexa Fluor 555-labeled Dodeca probe on the right arm of chromosome *3*, the samples were denatured at 92°C and hybridization was at 37°C. Heterochromatic probes were made by Integrated DNA Technologies. Samples with BAC probes to euchromatic regions of the *X* chromosome were denatured at 92°C and hybridization was carried out at 37°C. BAC probes for 3C (BACR03D13) and 7DE (BACR39F18) were labeled with ARES Alexa Fluor 488 or 647 DNA labeling kits (Invitrogen). BAC DNA was digested with *Alu*I, *Hae*III, *Mse*I, *Msp*I, *Rsa*I and *Mbo*I. Fragments were precipitated and then resuspended in water. The DNA was then denatured at 100°C for 1 minute and chilled immediately on ice. 20 µl of 5X terminal deoynucleotidyl transferase buffer, 20 µl of 25 mM Cobalt(II) chloride, 2.5 µl of 2 mM amionally dUTP from the ARES DNA labeling kit, 5 µl of 1 mM unlabeled 2′-deoxythymidine 5′-triphosphate, and 1 µl (400 units) Terminal deoxynucleotidyl Transferase (Roche) were added to 51.5 µl of the BAC DNA at room temperature. The reaction was allowed to proceed for 2 hr at 37°C. DNA was precipitated and the dried pellet resuspended in water. DNA was mixed with Ares labeling kit buffer and Ares dye that had been dissolved in dimethylsulfoxide. Sample was incubated in the dark for 1–2 hr at room temperature. The reaction was quenched with 1 M hydroxylamine. Probe was purified with a Qia-quick column (Qiagen). Labeled DNA was pelleted, allowed to dry and resuspended in water. Immuno-FISH was carried out as described [28].

For analysis of fixed ovaries, the DeltaVision microscopy system was used (Applied Precision, Issaquah, WA). The system is equipped with an Olympus 1X70 inverted microscope and high-resolution CCD camera. The images were deconvolved using the SoftWoRx v.25 software (Applied Precision). Embryos were imaged using an LSM-510 META confocal microscope (Zeiss) with a Plan-APO 40X objective (1.3 NA) with a zoom of 0.7 or a 63X Plan-apochromat (1.4 NA) with a zoom of 2. Images were acquired using the AIM software v4.2 by taking a Z stack and transformed into 2D projections using AIM software v4.2.

### Creation of the mutated RNAi construct

The sequence targeting the *Top2* gene is CACGAAGATATCCAACTACAA (top) and TTGTAGTTGGATATCTTCGTG (bottom). This 21-bp oligo matched a sequence of *CG33296* at 18/21 nucleotides. Oligos were created by Operon Biotechnologies Inc. to change the three differing nucleotides to precisely match *CG33296*. The 21-bp sense and anti-sense targeting sequences were CTCGAAGATATCCAACTTTAA and TTAAAGTTGGATATCTTCGAG. Oligos were annealed and ligated into a Valium22 vector that was digested with *Nhe*I and *EcoR*I according to the instructions provided by TRiP. Ligated product was transformed into competent cells. Genetic Services, Inc injected purified vector into *y sc v; attP2* flies and *y+ v+* flies were selected and sequence-verified.

### Hatch rate assays

Numerous females and males were allowed to acclimate to grape plates with yeast paste. Flies were transferred to fresh grape plates with yeast paste and females were allowed to lay eggs for 1–3 hours. Eggs within a grid on the plates were scored for hatching approximately 48 hr later.

### Live imaging

Live imaging was performed on prometaphase I oocytes as described [6]. Oocytes were injected using standard microinjection procedures with an approximately 1∶1 ratio of porcine rhodamine-conjugated α-tubulin minus glycerol (Cytoskeleton) and Quant-iT OliGreen ssDNA Reagent (Invitrogen) diluted 0.7 fold with water. Oocytes were imaged using an LSM-510 META confocal microscope (Zeiss) with an alpha plan-fluar 100X (1.4 NA) objective and a 1.5 zoom. Images were acquired using the AIM software v4.2 by taking a 10-series Z stack at 1 micron intervals with 20 seconds between acquisitions, which resulted in a set of images approximately every 45 seconds. Images were transformed into 2D projections and concatenated into videos using the AIM software v4.2.

### Western blot analysis

For each genotype, ovaries from virgin, yeast-fed females were dissected in cold 1X PBS, the ovaries were teased apart, and stage 14 oocytes were selected over the course of 2 hr. Stage 14 oocytes were homogenized in 50 µL of cold lysis buffer containing 150 mM NaCl, 50 mM Tris (pH 6.8), 2.5 mM EDTA, 2.5 mM EGTA, 0.1% Triton-X, and protease inhibitor cocktail (Sigma-Aldrich). Ovary lysates were cleared by centrifugation twice at 14,000 rpm for 15 min at 4°C. Lysates were assayed by Bradford and concentrations adjusted before samples were combined with 2X SDS sample buffer and boiled for 5 min, and the solubilized proteins were analyzed by Western blot using standard techniques. The primary antibody used for Western blot was rabbit anti-Top2 [21] at a dilution of 1∶5000 and α-tubulin (Serotec) at a dilution of 1∶5000. Immunoreactivity was detected using an alkaline phosphatase-conjugated rabbit secondary antibody (Jackson ImmunoResearch) and the nitroblue tetrazolium and 5-bromo-4-chloro-3-indolyl phosphatase (NBT/BCIP, Invitrogen) reagents.

## Supporting Information

Figure S1 Top2 levels are reduced in *Top2* RNAi/*matαGAL* oocytes compared to control oocytes. Westerns were probed with antibodies recognizing Top2 (top band) and α-tubulin (bottom band). Two independent samples of *Top2* RNAi*/matαGAL* oocytes are shown with oocytes from three sets of control flies: *y w; spa^pol^*, *Top2* RNAi*/+*, and *matαGAL/+*.
(TIF)

Figure S2 The defect in the separation of the heterochromatic region of the *X* chromosome is not due to a failure in spindle assembly. DNA is labeled with DAPI (blue), a FISH probe targeting the 359-bp repeat predominantly on the *X* chromosome is in green, and the spindle is labeled with an antibody recognizing α-tubulin (red). (A) *Top2* RNAi*/+* oocyte with properly bioriented *X* chromosomes on a bipolar spindle. (B–C) Examples of *Top2* RNAi*/matαGAL* oocytes with bipolar spindles, but the 359-bp region of the *X* chromosomes failed to separate. Images are projections of partial Z-stacks. Scale bars are 5 microns.
(TIF)

Figure S3 *CG33296* RNAi/*matαGAL* oocytes display normal chromosome morphology and biorientation. (A–C) Shown are *CG33296* RNAi/*matαGAL* oocytes. The *CG33296* RNAi construct was constructed by mutating the *Top2* RNAi sequence at three nucleotides. DNA is labeled with DAPI (blue) in all images. (A) Chromatin is labeled with an antibody recognizing H3S10p (green) and the spindle is labeled with an antibody recognizing α-tubulin (red). Shown is a bipolar spindle with *4^th^* chromosomes that have moved towards the poles. (B) A FISH probe targeting the 359-bp repeat predominantly on the *X* chromosome is in green and a FISH probe to the AATAT heterochromatic repeat on the *4^th^* chromosome and a minor repeat on the *X* chromosome is shown in red. The *X* and *4^th^* chromosomes are properly bioriented. (C) A FISH probe targeting the AACAC repeat on the right arm of the *2^nd^* chromosome is in green and a FISH probe to the Dodeca heterochromatic repeat on the right arm of the *3^rd^* chromosome is shown in red. The heterochromatic regions of *2R* and *3R* have properly separated and bioriented. Images are projections of partial Z-stacks. Scale bars are 5 microns.
(TIF)

Figure S4 Euchromatic regions can separate during prophase in *Top2* RNAi*/matαGAL* oocytes. DNA is labeled with DAPI (blue), a BAC FISH probe of 7DE is in green, and a BAC FISH probe of 3C is in red. Oocytes shown were approximately stages 9–10. (A) *Top2* RNAi*/+* control oocyte with two foci for both probes indicating these euchromatic regions have separated. (B) *Top2* RNAi*/+* control oocyte with two foci for the probe to the 7DE region indicating this euchromatic region had separated. Only one focus is present for the 3C region. (C–E) *Top2* RNAi*/matαGAL* oocytes displaying separation of euchromatic regions during mid-prophase. The 3C region is separated in (C–E) while there are two foci of the 7DE probe in (D). Images are projections of partial Z-stacks. Scale bars are 5 microns.
(TIF)

Figure S5 Chromatin threads can be observed in oocytes that fail to undergo recombination. Shown are prometaphase I oocytes dissected under non-activating conditions from *y; mei-W68^Z1049^ cn bw/mei-W68^Z4572^ cn bw* mothers. DAPI is labeled in blue, H3S10p is labeled in green, and α-tubulin is in red. Arrowheads point to chromatin threads connecting multiple DNA masses. Scale bars are 5 microns.
(TIF)

Figure S6 Females expressing the *Top2* RNAi construct with the nanos-Gal4:VP16 driver fail to produce normal ovaries. Ovaries at top are from a *y w; spa^pol^* female while the ovaries at the bottom are from a mother expressing the *Top2* RNAi construct with the nanos-Gal4:VP16 driver that starts expressing early in the ovary [53]. Knocking down *Top2* in the early stage of the ovaries led to a cessation of ovarian development.
(TIF)

Video S1 Spindle assembly occurs normally in a living *Top2* RNAi*/matαGAL* oocyte. Shown is a time-lapse of a living *Top2* RNAi*/matαGAL* oocyte. DNA is labeled in white and α–tubulin is labeled in blue. After germinal vesicle breakdown, a bipolar spindle quickly forms. Bipolarity is maintained for the remainder of imaging and achiasmate *4^th^* chromosomes fail to initiate prometaphase I movements. Video is a projection from Z-stacks and is presented at 5 frames per second.
(MP4)
