# Supplementary figures and images for: Topoisomerase II Is Required for the Proper Separation of Heterochromatic Regions during Drosophila melanogaster Female Meiosis
Source: PLoS Genet. 2014 Oct 23;10(10):e1004650. doi: 10.1371/journal.pgen.1004650 (PMC4207608; doi:10.1371/journal.pgen.1004650)

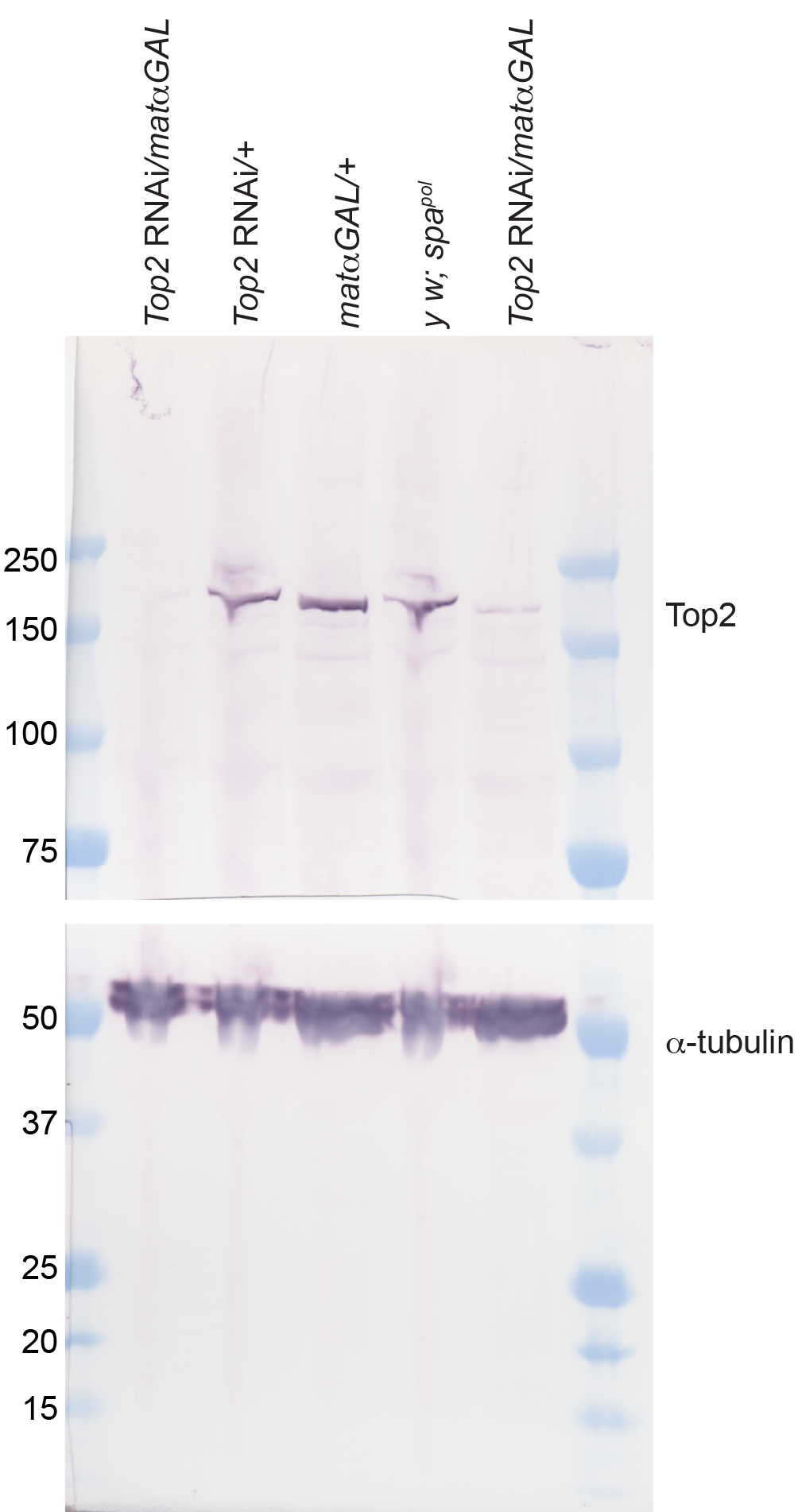

Supplement: Figure S1 — Top2 levels are reduced in Top2 RNAi/matαGAL oocytes compared to control oocytes. Westerns were probed with antibodies recognizing Top2 (top band) and α-tubulin (bottom band). Two independent samples of Top2 RNAi/matαGAL oocytes are shown with oocytes from three sets of control flies: y w; spapol, Top2 RNAi/+, and matαGAL/+. (TIF) [file pgen.1004650.s001.tif]

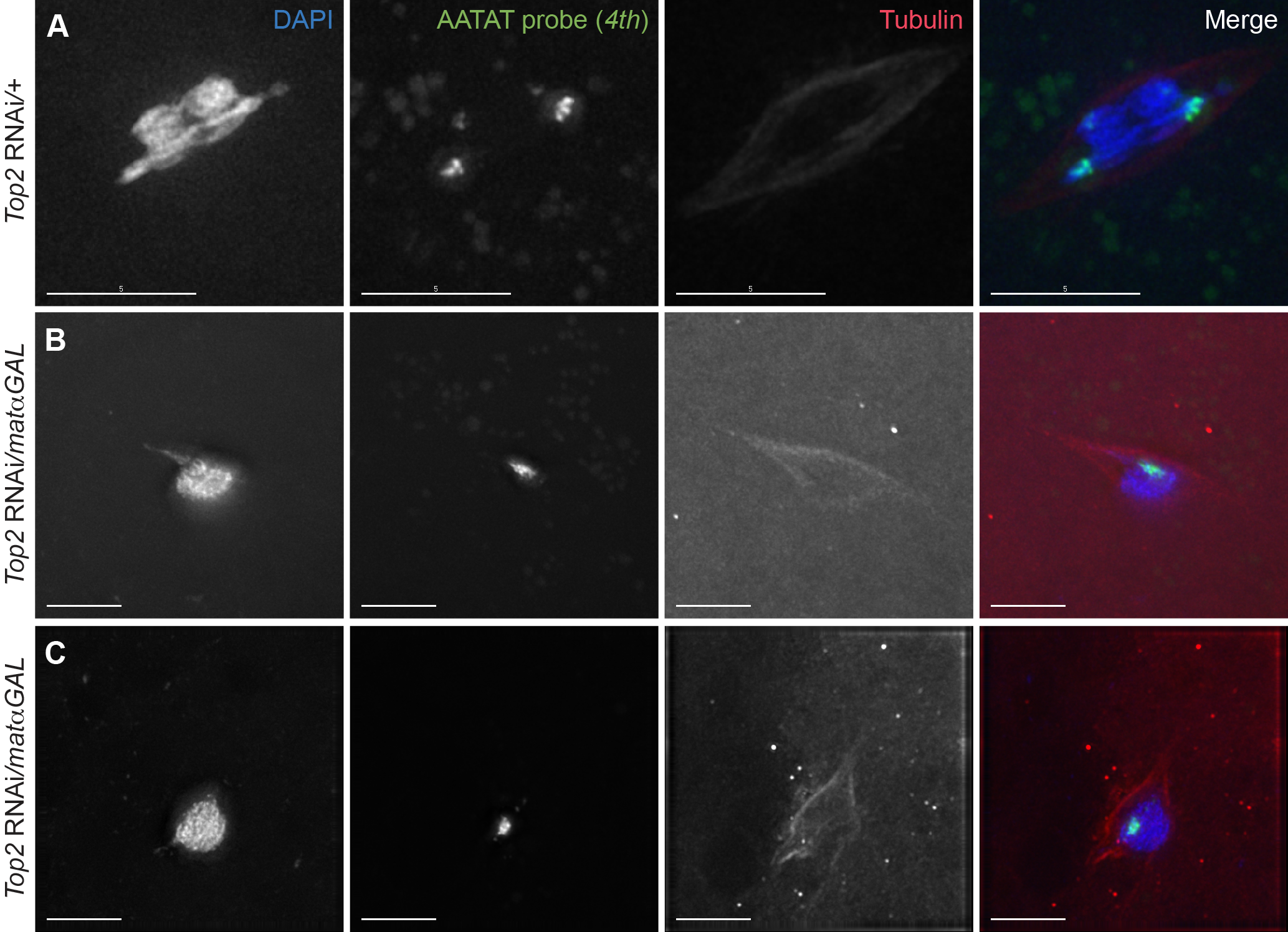

Supplement: Figure S2 — The defect in the separation of the heterochromatic region of the X chromosome is not due to a failure in spindle assembly. DNA is labeled with DAPI (blue), a FISH probe targeting the 359-bp repeat predominantly on the X chromosome is in green, and the spindle is labeled with an antibody recognizing α-tubulin (red). (A) Top2 RNAi/+ oocyte with properly bioriented X chromosomes on a bipolar spindle. (B–C) Examples of Top2 RNAi/matαGAL oocytes with bipolar spindles, but the 359-bp region of the X chromosomes failed to separate. Images are projections of partial Z-stacks. Scale bars are 5 microns. (TIF) [file pgen.1004650.s002.tif]

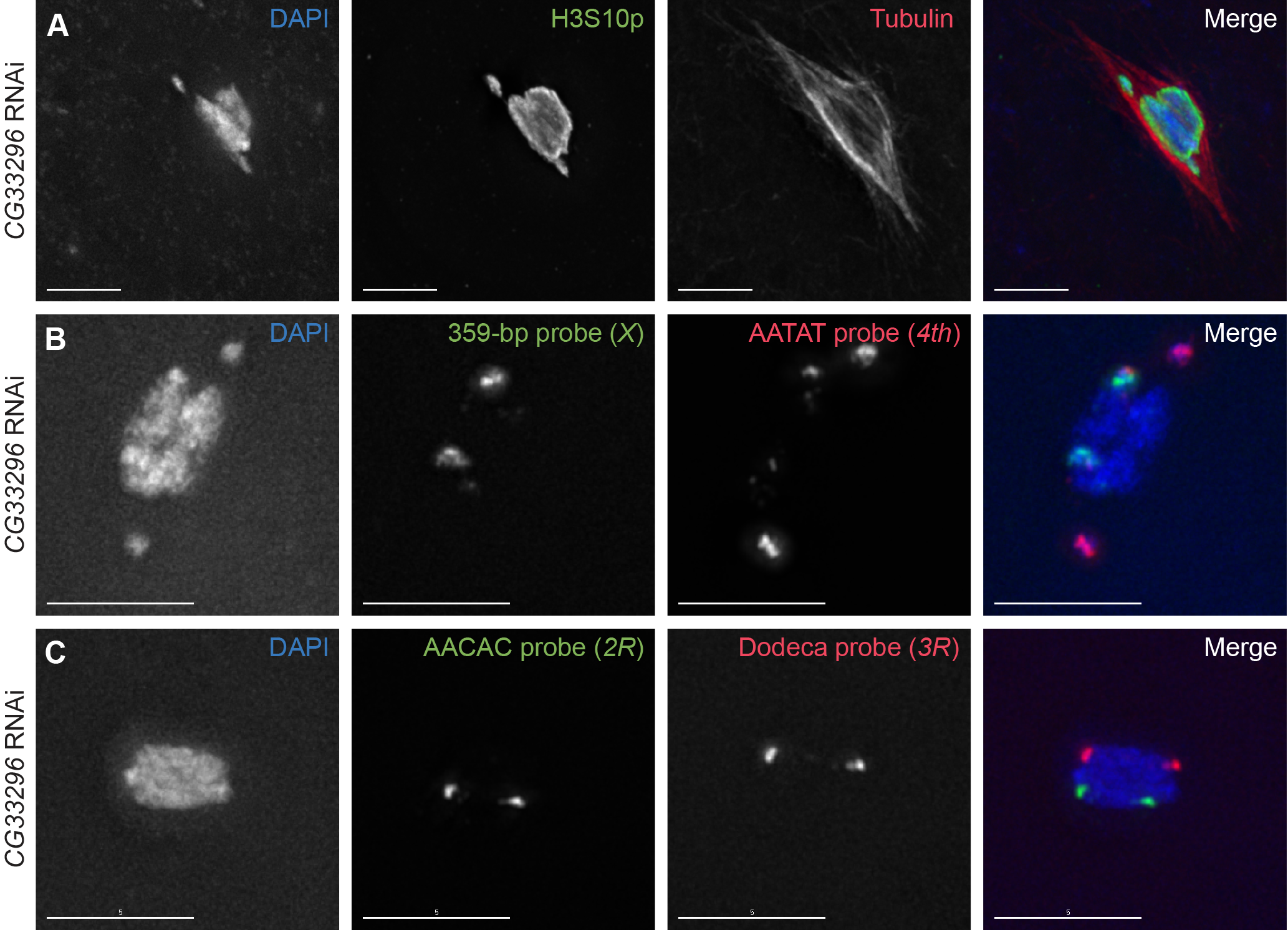

Supplement: Figure S3 — CG33296 RNAi/matαGAL oocytes display normal chromosome morphology and biorientation. (A–C) Shown are CG33296 RNAi/matαGAL oocytes. The CG33296 RNAi construct was constructed by mutating the Top2 RNAi sequence at three nucleotides. DNA is labeled with DAPI (blue) in all images. (A) Chromatin is labeled with an antibody recognizing H3S10p (green) and the spindle is labeled with an antibody recognizing α-tubulin (red). Shown is a bipolar spindle with 4th chromosomes that have moved towards the poles. (B) A FISH probe targeting the 359-bp repeat predominantly on the X chromosome is in green and a FISH probe to the AATAT heterochromatic repeat on the 4th chromosome and a minor repeat on the X chromosome is shown in red. The X and 4th chromosomes are properly bioriented. (C) A FISH probe targeting the AACAC repeat on the right arm of the 2nd chromosome is in green and a FISH probe to the Dodeca heterochromatic repeat on the right arm of the 3rd chromosome is shown in red. The heterochromatic regions of 2R and 3R have properly separated and bioriented. Images are projections of partial Z-stacks. Scale bars are 5 microns. (TIF) [file pgen.1004650.s003.tif]

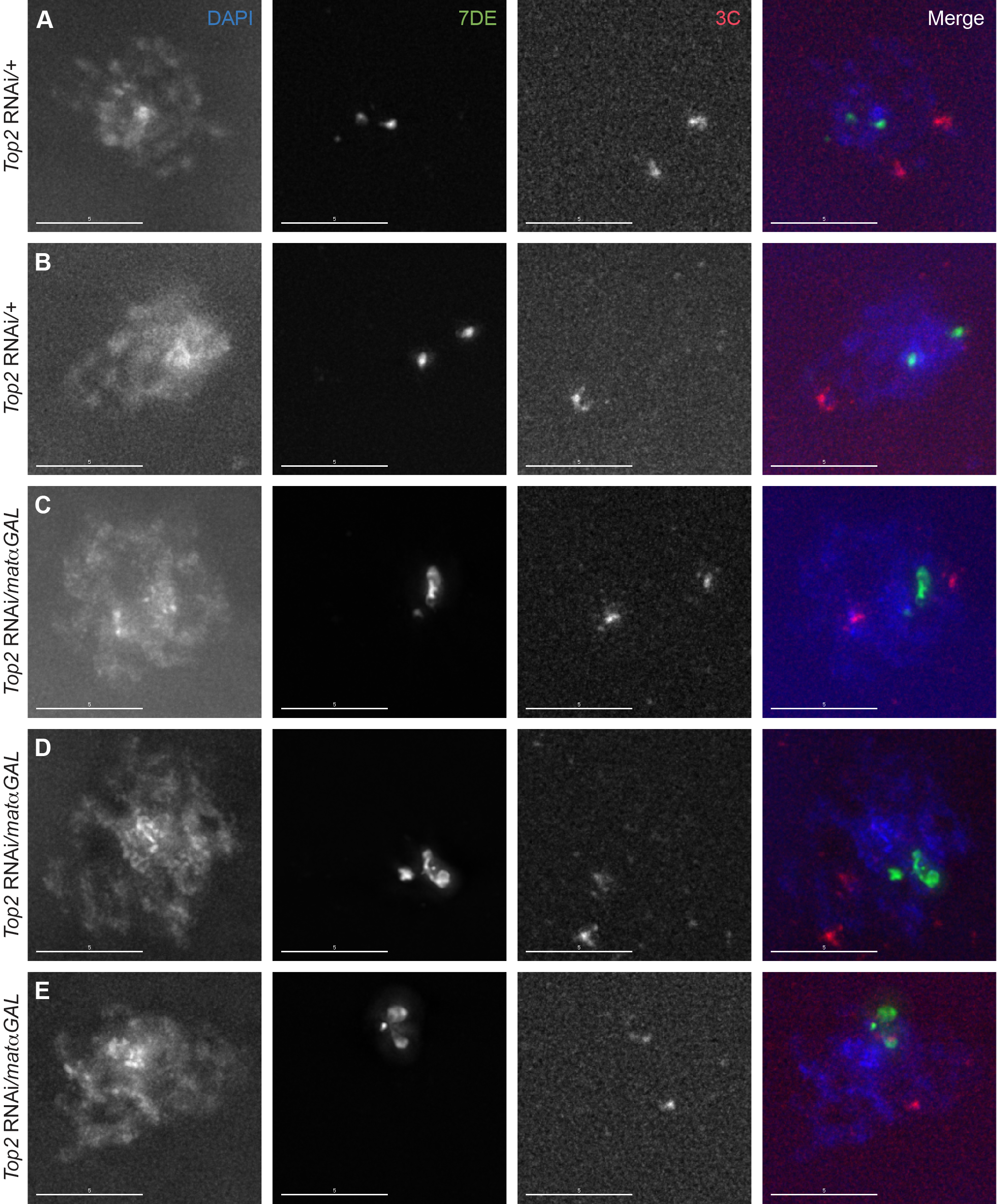

Supplement: Figure S4 — Euchromatic regions can separate during prophase in Top2 RNAi/matαGAL oocytes. DNA is labeled with DAPI (blue), a BAC FISH probe of 7DE is in green, and a BAC FISH probe of 3C is in red. Oocytes shown were approximately stages 9–10. (A) Top2 RNAi/+ control oocyte with two foci for both probes indicating these euchromatic regions have separated. (B) Top2 RNAi/+ control oocyte with two foci for the probe to the 7DE region indicating this euchromatic region had separated. Only one focus is present for the 3C region. (C–E) Top2 RNAi/matαGAL oocytes displaying separation of euchromatic regions during mid-prophase. The 3C region is separated in (C–E) while there are two foci of the 7DE probe in (D). Images are projections of partial Z-stacks. Scale bars are 5 microns. (TIF) [file pgen.1004650.s004.tif]

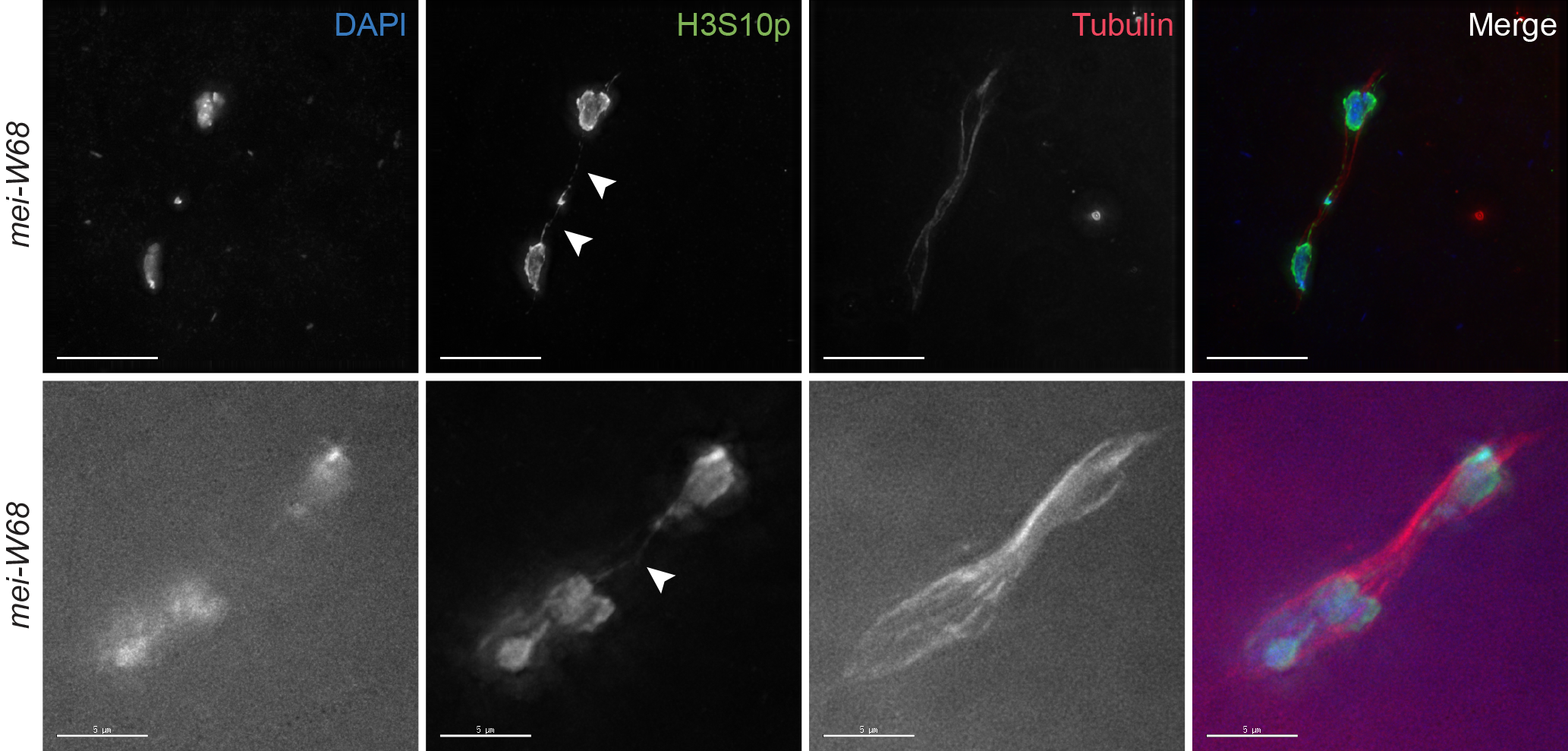

Supplement: Figure S5 — Chromatin threads can be observed in oocytes that fail to undergo recombination. Shown are prometaphase I oocytes dissected under non-activating conditions from y; mei-W68Z1049 cn bw/mei-W68Z4572 cn bw mothers. DAPI is labeled in blue, H3S10p is labeled in green, and α-tubulin is in red. Arrowheads point to chromatin threads connecting multiple DNA masses. Scale bars are 5 microns. (TIF) [file pgen.1004650.s005.tif]

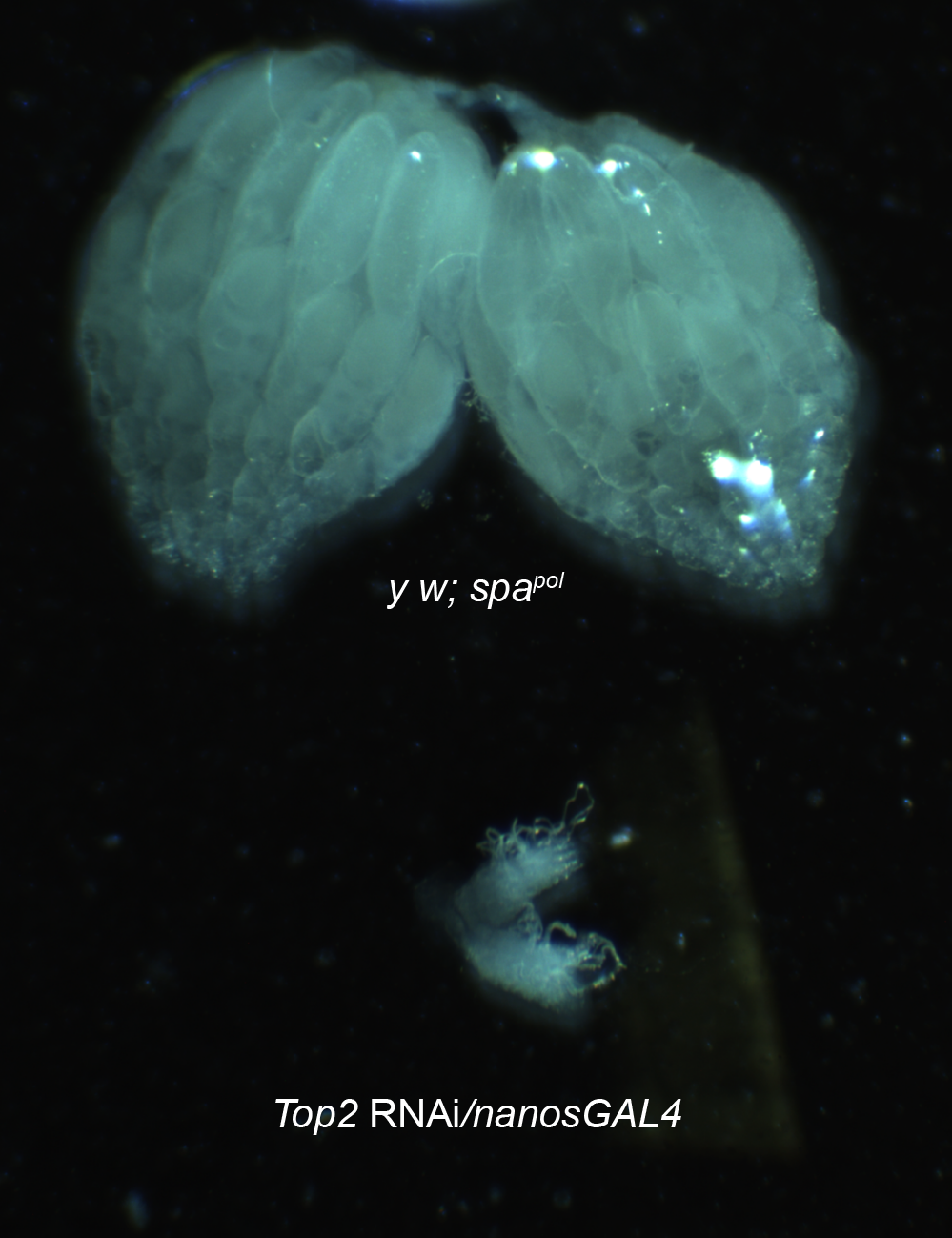

Supplement: Figure S6 — Females expressing the Top2 RNAi construct with the nanos-Gal4:VP16 driver fail to produce normal ovaries. Ovaries at top are from a y w; spapol female while the ovaries at the bottom are from a mother expressing the Top2 RNAi construct with the nanos-Gal4:VP16 driver that starts expressing early in the ovary [53]. Knocking down Top2 in the early stage of the ovaries led to a cessation of ovarian development. (TIF) [file pgen.1004650.s006.tif]
